# The Unequal World of Health Data

**DOI:** 10.1371/journal.pmed.1000155

**Published:** 2009-11-24

**Authors:** Peter Byass

**Affiliations:** Umeå Centre for Global Health Research, Department of Public Health and Clinical Medicine, Umeå University, Umeå, Sweden

## Abstract

Peter Byass argues that less data are available on the health of the poor than of the rich, and discusses several alternative strategies to improve the representativeness of health data.

**Summary Points**

- Health data, poverty, and inequality exist in a complex global co-dependency, therefore making meaningful comparisons of health across widely different settings challenging.
- Less data exist on the health of the poor than of the rich, which in turn raises important questions as to how representative available data are in relation to populations that go uncounted.
- Alternative strategies are needed to fill in inequitable gaps in data.
- Poverty either in physical terms or in data does not justify the use of impoverished research methods or ethical standards. Reasonable, realistic, and contextually appropriate approaches to research are needed.

## Health Data

Health data are a slippery commodity. It is not difficult to reach subjective agreement that such data are unequally distributed around the world, both in terms of quantity and quality. But objective assessments are more difficult, since they need to be based on data—and the data about the data may themselves be problematic or incomplete. Perhaps not surprisingly, therefore, literature is relatively scarce on the extent and quality of primary sources of health data on a global scale, even though there are many reports that present aggregated global data on various health issues [1],[2], sometimes giving the impression that those estimates carry a high degree of certainty.

## Health Data, Information, Knowledge, and Publications

Having established that the quality of health data has contextual determinants, what are the implications for the global medical literature? Health data essentially lie at the bottom of a continuum that moves up through information, knowledge, and publications (publications in the medical literature and evidence-based policy initiatives somehow representing “wisdom”) [3]. The concept of the “10/90 gap,” which characterises global disparity in terms of only 10% of research effort and resources being directed towards the 90% of need located among the globally disadvantaged, has largely been justified via bibliometric and research funding studies [4]–[8], rather than in terms of data. This is not particularly surprising, given the relative ease of quantifying publications globally via databases such as PubMed (www.pubmed.com), and analysing such data by place of origin. Bibliometric parameters may to some extent act as valid proxies for describing underlying sources of health data, but such links should not be accepted unquestioningly. Many health data are collected routinely without any intention of carrying out research, particularly in poorer settings, but may be just as important and valid as those that underlie the completed and published research counted bibliometrically.

It is also the case that the overall volume of health-related data is increasing exponentially, while the number of publications is advancing more linearly [9], and so data-to-publication relationships are in a continual state of change. Many health data never contribute to higher level outputs, but their inclusion in or exclusion from analytic processes are not necessarily related to their potential impact nor to the magnitude of health issues to which they pertain. It is interesting to see medical journals, most recently *PLoS Medicine* [10], more explicitly targeting subject matter linked to high-magnitude issues, the extent of which are revealed by analyses of health data.

## Health Data and Poverty

**Figure 1:**
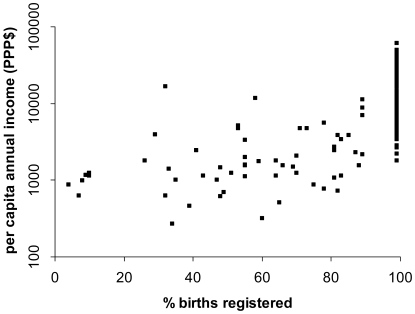
Proportion of births registered versus per capita annual income (log scale) for 151 countries with available data (http://www.who.int/whosis).

Poverty in material terms is inextricably linked with poverty of data. Figure 1 shows as an example country data for per capita annual income and percentage of births registered, using WHOSIS data (www.who.int/whosis). Of 193 countries, 42 had missing data, and 94 (with an income range of purchasing power parity dollars [PPP$] 1,790 to PPP$60,870, with a mean of PPP$17,357) claimed virtually complete birth registration. By comparison, the 57 countries reporting lower levels of birth registration had an income range of PPP$270 to PPP$16,620, and a mean of PPP$2,675. If records of something as fundamental to health as individual birth registration data are so strongly dependent on economic status, questions have to be asked about the coverage and quality of other health data in poorer settings.

If incompleteness of data is a poverty-linked problem, then the representativity of those data that do exist becomes a crucial issue. This is especially true for data originating from health facilities, which depend critically on the self-selected population subgroup using a particular facility. One way of addressing these issues in settings that are unlikely to have complete coverage at high quality in the near future is to select a circumscribed population from which reasonably detailed, complete, and quality community-based data can be gathered longitudinally, such as is done by Health and Demographic Surveillance Sites (HDSS) within the Indepth Network [11]. Valid questions still arise as to how representative such sites may be of surrounding areas, but to some extent this can be checked by comparing results for key parameters with other kinds of survey data, such as national censuses or Demographic and Health Survey (DHS) data [12]. All of these strategies are to some extent compromises, compared with a commitment to detailed, on-going registration and surveillance of all individuals within a national population, such as has been practised, for example, for more than two centuries in Sweden [13]. However, in countries where population-level services are highly resource-constrained, it is unreasonable to expect universal population-based registration to be implemented ahead of other more pressing priorities, and hence interim strategies such as the implementation of HDSSs are very important.

## A Broad View of “Health” Data

So far, I have implied a relatively conventional view of health data, thinking in terms of data on patient treatment, disease occurrence, and similar entities. When applied to populations rather than facilities, demographic parameters have to be included in this scope—but, at least in richer parts of the world, these are often routinely available from civic data. However, the concept of health data can and should be widened further in order to understand what really goes on within populations. For example, major disease epidemics and mortality peaks in a rural Ethiopian community turned out to be explained by quite subtle variations in the seasonal patterns of rainfall [14]. Fortunately in this case, rainfall in the locality had been routinely recorded by official meteorological services, and post hoc analyses were possible.

Some degree of lateral thinking is sometimes needed to make connections between available data and population health. A particularly imaginative example of this type of thinking establishes correlations between night-time light, as seen by satellites, and poverty across Africa [15]. This analysis clearly shows that something as seemingly obscure as satellite data on night-time light are indeed valuable health data—and have the advantage of being available and regularly updated for the entire African continent, something that cannot be said for many more conventional sources of health data.

Complementary sources of health data are likely to become increasingly relevant as the spectre of climate change emerges with increasing importance in relation to some of the world's most vulnerable populations, for example in sub-Saharan Africa.

## Health Data, Poverty, and Methods

If the quantity and quality of available health data, and hence of scientific manuscripts, are partly determined by poverty, then important questions arise as to how research findings from relatively impoverished settings can find their way into the global literature. While few would argue for blatant resource-dependent double standards in research methods or ethics, there is a delicate balance to be achieved between what is contextually good enough, feasible, and appropriate—with idealised and unattainable approaches falling away to one side, and “quick and dirty” techniques falling away on the other. Failing to reach the right balance here creates a two-way danger of bias: either publishing substandard research from poor settings because nothing better is available, or excluding results from poor settings from the literature because nothing is offered of sufficient quality.

An interesting methodological example illustrating this situation is around cause-of-death registration, concerning data that have long been considered essential for understanding population health and disease transition. In large parts of the world there are long-standing traditions of physician certification of death, usually made compulsory via agencies outwith the health sector. In other large parts of the world, deaths may not even be counted, let alone certified as to cause—and of course there is a substantial poverty gradient across these extremes.

In response to the paucity of cause-of-death data in the developing world, verbal autopsy (VA) methods have emerged in recent decades (in which data on circumstances of death are gathered during an interview with family or friends after the death, and subsequently interpreted as to cause) [16]. With the benefit of hindsight, one can see that much of the earlier work on developing VA methods was based on an unstated assumption that the aim was to emulate physician certification using what might be a second-best method. Accordingly, very little critical attention has been given in VA work to the shortcomings of routine physician death certification, with a tendency to take physician reports of cause-of-death as a “gold standard.” The increasingly clear conclusion is that cause-of-death work on a global scale has to be based on a combination of physician certificate and VA data, in which the advantages and disadvantages of both methods are clearly discussed and understood, and in which any correlations between cause-of-death and poverty are very carefully interpreted in the light of the confounding between poverty and methodology.

And finally, there is also a need for a more honest debate as to how good is good enough in terms of health data. Many data gathering operations make the implicit assumption that their data should be 100% correct, even though most experienced data managers would acknowledge that this is an unrealistic ideal, particularly in poor environments. They would also be quick to agree that a large amount of their time and resources are spent on cleaning and correcting relatively small proportions of incorrect or missing data. But how many health policies have been wrongly implemented as a consequence of data errors? Unless data errors are made on a very large scale, or on a highly systematic (i.e., nonrandom) basis, they are unlikely to lead to reversals on conclusions of public health significance [17].

## Conclusion

Perhaps this Essay raises as many questions as it provides answers across this complex and sometimes dangerous mix of health data, poverty, and inequality. My hope in presenting this material is that it may help readers to understand some of the underlying issues when interpreting published work from around the world, which of necessity draws on multifarious health data from widely different settings.

## Abbreviations

VA: verbal autopsy
